# Hepatobiliary Complications in Children with Sickle Cell Disease: A Retrospective Review of Medical Records from 616 Patients

**DOI:** 10.3390/jcm8091481

**Published:** 2019-09-18

**Authors:** Slimane Allali, Mariane de Montalembert, Valentine Brousse, Claire Heilbronner, Melissa Taylor, Josephine Brice, Elisabetta Manzali, Nicolas Garcelon, Florence Lacaille

**Affiliations:** 1 Department of General Pediatrics and Pediatric Infectious Diseases, Necker-Enfants malades Hospital, Assistance Publique-Hôpitaux de Paris (AP-HP), Université de Paris, 75005 Paris, France; slimaneallali@hotmail.fr (S.A.); melissa.taylor@aphp.fr (M.T.); josephine.brice@aphp.fr (J.B.); 2 Laboratory of Cellular and Molecular Mechanisms of Hematological Disorders and Therapeutical Implications, Paris Descartes-Sorbonne Paris Cite University, Imagine Institute, U1163 Inserm, France; 3 Laboratory of Excellence GR-Ex, 75015 Paris, France; valentine.brousse@gmail.com; 4 Pediatric Intensive Care and High Dependency Unit, Necker-Enfants malades Hospital, Assistance Publique-Hôpitaux de Paris (AP-HP), Université de Paris, 75005 Paris, France; claire.heilbronner@aphp.fr; 5 Department of Pediatric Gastroenterology-Hepatology-Nutrition, Hepatology Unit, Necker-Enfants malades Hospital, Assistance Publique-Hôpitaux de Paris (AP-HP), Université de Paris, 75005 Paris, France; elisabetta.manzali@gmail.com (E.M.); florence.lacaille@aphp.fr (F.L.); 6 Data science Platform, Paris Descartes—Sorbonne Paris Cite University, Imagine Institute, U1163 Inserm, France; nicolas.garcelon@institutimagine.org

**Keywords:** sickle cell hepatopathy, cholelithiasis, acute hepatic crisis, cholangiopathy

## Abstract

Hepatobiliary complications in children with sickle cell disease (SCD) are rarely reported but can be life-threatening. We retrospectively assessed their prevalence in a cohort of 616 children followed in a French university-hospital SCD reference center. Eligibility criteria were the following: age <18 years, seen at least twice with an interval of more than 6 months from January 2008 to December 2017, with all genotypes of SCD. Patients with hepatobiliary complications were identified via the local data warehouse and medical files were thoroughly reviewed. At least one hepatobiliary complication was reported in 37% of the children. The most frequent was cholelithiasis, in 25% of cases, which led to systematic screening and elective cholecystectomy in the case of gallstones. Overall, 6% of the children experienced acute sickle cell hepatic crisis, sickle cell intra-hepatic cholestasis, or acute hepatic sequestration, with severity ranging from mild liver pain and increased jaundice to multiple organ failure and death. Emergency treatment was exchange transfusion, which led to normalization of liver tests in most cases. Five children had chronic cholangiopathy, associated with auto-immune hepatitis in two cases. One needed liver transplantation, having a good outcome but with many complications. Transfusion iron load and infectious hepatitis cases were mild. Hepatotoxicity of an iron chelator was suspected to contribute to abnormal liver test results in five patients. We propose recommendations to prevent, explore, and treat hepatobiliary complications in SCD children. We underline the need for emergency exchange transfusion when acute liver failure develops and warn against liver biopsy and transplantation in this condition.

## 1. Introduction

Sickle cell disease (SCD) is one of the most frequent genetic diseases in the world [1]. Sickle cell hepatopathy, a generic name for liver complications in SCD, includes mild biochemical abnormalities, frequent biliary lithiasis, and rare but potentially lethal complications, such as liver failure during acute hepatic crisis [2,3,4].

SCD is characterized by the presence of abnormal hemoglobin S (HbS), either in homozygous status (HbSS), or combined with another abnormal hemoglobin (Hb), the most frequent combinations being HbSC and HbS/β-thalassemia. Complications arise from the shape change of erythrocytes on deoxygenation, secondary to polymerization of the abnormal HbS. Sickled erythrocytes cause vaso-occlusion together with abnormal endothelial interactions, leading to repeated ischemia, ischemia/reperfusion injuries, inflammation, and endothelial dysfunction. All organs may be affected, the frequency of organ dysfunctions increasing with age [5].

**Figure 1:**
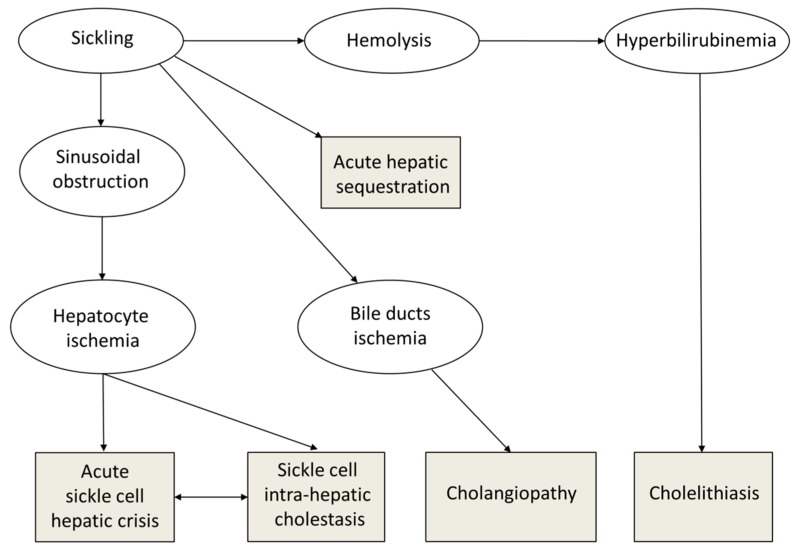
Pathophysiology of hepatobiliary complications in sickle cell disease (SCD). Sinusoidal obstruction by sickled red blood cells results in hepatocyte ischemia, with secondary ballooning of adjacent hepatocytes and intracanalicular cholestasis. Vascular obstruction may also cause red cell and platelet trapping in the liver, leading to acute hepatic sequestration. Depending on the relative degrees of ischemia, cholestasis, and cell trapping, the crises may present as acute sickle cell hepatic crisis, sickle cell intra-hepatic cholestasis, or hepatic sequestration. Bile ducts ischemia secondary to sickling is responsible for cholangiopathy, and chronic hemolysis is responsible for hyperbilirubinemia, promoting cholelithiasis formation.

The pathophysiology of hepatobiliary complications in SCD is complex (Figure 1). Sinusoidal obstruction, due to sickled red cells and Kupffer cell erythrophagocytosis and hyperplasia, is responsible for hepatocyte ischemia, with secondary ballooning of adjacent hepatocytes and intracanalicular cholestasis [2,3,6]. Thus, acute liver failure during hepatic crisis in SCD could be compared to hypoxic hepatitis complicating cardiogenic shock, which recovers quickly if the cause is treated. The vascular obstruction may also cause red cell and platelet trapping, called “sequestration”, in the liver or spleen (Figure 1). Depending on the relative degrees of ischemia, cholestasis, and cell trapping, the crises may present as acute sickle cell hepatic crisis, sickle cell intrahepatic cholestasis, or hepatic sequestration. However, these are probably different presentations of the same phenomenon. Recurrent crises and chronic or repeated ischemia are responsible for the lesions seen in adults, sinusoidal fibrosis, focal necrosis, portal fibrosis, nodular regenerative hyperplasia, and cirrhosis, contributing to the chronic debilitating disease of adult patients with SCD [2]. Furthermore, chronic hemolysis induces hyperbilirubinemia, leading to increased bilirubin load in biliary ducts, and releases heme that might promote SCD liver damage by inducing proinflammatory activation of liver macrophages, as reported in an SCD mouse model [7]. Liver damage in SCD may also be due to iron overload, viruses, or autoimmune disorders.

Prevalence of liver dysfunction in adults with SCD is estimated to be around 10% and is expected to increase in the aging SCD population [6]. Acute manifestations are sickle cell hepatic crisis, sickle cell intrahepatic cholestasis, and hepatic sequestration. Chronic manifestations include cholelithiasis, sickle cell cholangiopathy, auto-immune hepatitis, viral hepatitis, and iron overload [2,3,4,6,8]. In children, severe liver disease is rarely reported but might be underdiagnosed and insufficiently treated, because of misinterpretation of liver tests and possible confusion between hemolysis and liver disease markers [9]. Large series are rare, reporting mild elevation of transaminases and cholelithiasis as the main hepatobiliary abnormalities [9], and most studies are case series [10,11,12]. Here we assessed the prevalence of hepatobiliary manifestations in our cohort of more than 600 patients. We delineate the frequency and severity of these complications, discuss their respective treatment, point to the awareness of urgent situations, and discuss the specific follow-up of these patients, hepatic complications being increasingly recognized as an important cause of morbidity and mortality in adults [6].

## 2. Materials and Methods

### 2.1. Study Design

We performed a retrospective observational study between January 2008 and December 2017 in a pediatric French university-hospital reference center for SCD, in Paris. Patients were identified via the local data warehouse [13]. Inclusion criteria were the following: age <18 years, seen at least twice in our hospital with an interval of more than 6 months (to exclude infants seen only once after newborn screening, for SCD diagnosis announcement before being followed in another hospital), with all genotypes of SCD (HbSS, HbSC, HbS/β-thalassemia). We obtained 616 patient records and searched for the following keywords: hepatic complication, hepatic cytolysis, cholestasis, cholelithiasis, cholecystitis, gallstone, cholangitis, cholangiopathy, bile drainage, biliary tube, acute sickle cell hepatic crisis, acute hepatic sequestration, acute intrahepatic cholestasis, autoimmune hepatitis, liver failure, liver dysfunction, cholecystectomy, and viral hepatitis. Among records for 297 patients with at least one keyword identified, 68 were excluded, 48 because of selection error (the file said “no liver event”), 3 because abnormalities occurred after hematopoietic stem cell transplantation, and 17 because of a misdiagnosis (hemolysis and no liver disease). To better describe acute hepatic crisis and acute hepatic sequestration, which are rare and severe complications, we also analyzed data for 8 patients not regularly followed up in our hospital who were referred for management of their acute complication (6 acute hepatic crises and 2 acute hepatic sequestrations). Prevalence rates were calculated without these 8 patients. The final number of patients with hepatobiliary complications was 237. All medical records were thoroughly reviewed by M.d.M., and discussed by S.A., M.d.M. and F.L. if classification was uncertain. M.d.M. extracted the data; S.A. and F.L. reviewed the quality of data management. The main clinical and biological data obtained from patient medical files are listed in Table 1 and Table 2.

Our study was approved by the local medical ethics committee (n°20190806164057).

### 2.2. Classification of Hepatobiliary Complications

Complications were classified as follows:

- Biliary complications included cholelithiasis, cholecystitis, and cholangitis. Distinction was made between non-migrating lithiasis (some diagnosed after abdominal pain without radiological signs of migration) and migrating lithiasis diagnosed by ultrasonography.
- Cholangiopathy was defined as abnormal bile ducts with stenosis and dilations on ultrasonography and MR cholangiography, and elevated gamma-glutamyltransferase (γ-GT) level. Presence of antineutrophil cytoplasmic antibodies (ANCAs) or associated autoimmune hepatitis was classified as primary sclerosing cholangitis (PSC) [14].
- Auto-immune hepatitis was defined as elevated levels of transaminases (alanine aminotransferase (ALT) level more than twice the upper limit of normal) with hypergammaglobulinemia, positive autoantibodies (antinuclear antibodies (ANAs), anti-smooth muscle antibodies (SMAs), or anti-liver-kidney microsomes antibodies type 1 (LKM-1)), and compatible histopathology (portal inflammation and interface hepatitis).
- Acute sickle cell hepatic crisis was defined by the acute onset of right upper-quadrant abdominal pain, possibly associated with increased hepatomegaly and jaundice, with ALT level more than twice the upper limit of normal or elevated bilirubin level with predominantly conjugated fraction, with no other cause (virus, toxic, gallstone complication). Aspartate aminotransferase (AST) level was not considered for the diagnosis of liver disease because it depends also on hemolysis. We classified “sickle cell intrahepatic cholestasis” as the severe variant of acute hepatic crisis, with coagulopathy, and a possible evolution towards multiple organ failure, as previously published [6].
- Acute hepatic sequestration was defined by the sudden increase in liver size, associated with right upper-quadrant abdominal pain, and an acute decrease in Hb level >2 g/dL. Acute anemia was usually associated with thrombocytopenia and normal or increased reticulocyte count. Because it could be associated with increased transaminase levels, increased conjugated bilirubin level and liver failure, acute hepatic sequestration could also be considered a variant of acute hepatic crisis.
- Transfusion-related liver iron overload was diagnosed by markedly increased ferritin level with MRI-measured liver iron content (LIC) >3 mg iron/g dry weight.
- Hepatotoxicity of chelator drugs was evoked on abnormal liver tests after excluding other causes. Some patients underwent a reintroduction challenge: drug was reintroduced at a lower dose and liver tests were controlled.
- Infectious hepatitis was diagnosed on abnormal liver tests with the identification of a causative infectious agent (mainly viruses).
- Isolated biochemical abnormalities were defined as increased ALT level more than twice the upper limit of normal, elevated conjugated bilirubin level, or elevated γ-GT level, in the absence of any symptom.

### 2.3. Statistical Analysis

Data are expressed as median (range; interquartile range (IQR)), mean ± standard deviation (SD) or percentage. Differences between groups were assessed by Mann-Whitney test or Fisher’s exact test or chi-square test. *p* < 0.05 was considered statistically significant.

## 3. Results

**Figure 2:**
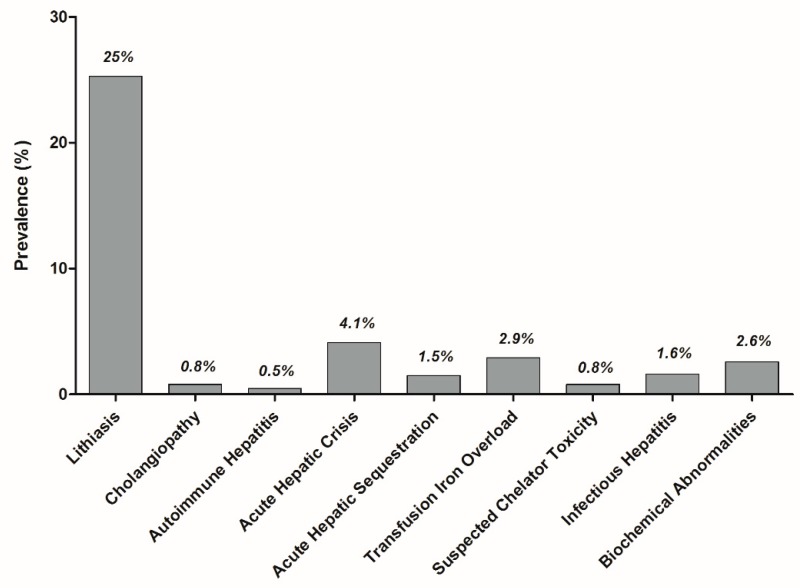
Prevalence of hepatobiliary complications in 616 children with sickle cell disease, followed up in our reference center from January 2008 to December 2017. “Acute hepatic crisis” includes acute sickle cell hepatic crisis and sickle cell intrahepatic cholestasis.

The prevalence of hepatobiliary complications is reported in Figure 2. At least one complication was experienced by 37% of the 616 children. The main clinical characteristics of the 237 patients with hepatobiliary complications are reported in Table 1. Hb level and liver test results are reported in Table 2.

### 3.1. Cholelithiasis

Gallstones were diagnosed in 156 of 616 (25%) children. Median Hb level at diagnosis was 8.4 g/dL, and prevalence of G6PD deficiency (16%) was not significantly higher than in other complication groups (*p* = 0.28). In 90 cases (58%), gallstones were incidentally found during the yearly check-up. Ultrasonography allowing gallstone identification was indicated for abdominal pain in 44 cases (28%) and for osteomyelitis in 3 cases (related twice to salmonella). Three children experienced cholecystitis. Gallstone migration was diagnosed by ultrasonography performed because of abdominal pain in 19 children (12%), complicated in one patient with pancreatitis and in three others with cholangitis. Gallstones were found in two children who had undergone a cholecystectomy 4 months and 16 years earlier.

Liver test results did not differ in children with asymptomatic gallstones or pain but showed a statistically significant elevation in γ-GT (*p* < 0.001), conjugated bilirubin (*p* = 0.001) and ALT (*p* < 0.001) levels during migration episodes (Table 2).

In 7 cases, the gallstone had disappeared on control ultrasonography. Two patients were lost to follow-up. The 147 remaining patients underwent elective cholecystectomy, combined in 12 cases with splenectomy because of hypersplenism or recurrent splenic sequestration.

### 3.2. Cholangiopathy

Prevalence of this complication was very low (Figure 2). In the five patients, bile duct dilation was suspected on ultrasonography and confirmed on MR-cholangiography. The dilation was secondary to a benign tumor of the pancreatic isthmus in one child. Two patients had autoimmune hepatitis and PSC (“overlap syndrome”) with ANAs, SMAs, and ANCAs, and one also had colitis. Another girl had a heterozygous mutation in the *MDR3* gene (homozygous mutation causing progressive familial intrahepatic cholestasis type 3). This patient and one of the two patients with overlap syndrome required temporary biliary drainage to control cholangitis. This second patient subsequently underwent Roux-en-Y biliary anastomosis, but uncontrolled fungal cholangitis developed. At age 9 years, she underwent liver transplantation, with many complications including sickle crises despite aggressive exchange transfusions, seizures and severe rejection. She was well 4 years later, with double immunosuppression, antiepileptic and antihypertensive treatment, monthly exchange transfusions, and subcutaneous daily deferoxamine. The last patient, a 10-year old girl, had dilation of intra- and extra-hepatic ducts with normal liver test results and no autoantibodies. Of note, the proportion of patients receiving hydroxyurea was the highest (80%) in this group (Table 1).

### 3.3. Auto-Immune Hepatitis

Three HbSS patients (0.5%) had auto-immune hepatitis. Two had overlap syndrome (*supra*): one underwent liver transplantation, and for the other patient, ulcerative pancolitis was controlled with prednisone, azathioprine, and ursodeoxycholic acid. In an 18-year-old boy, treatment with prednisone and azathioprine was successful. Immunosuppressive and steroid treatments were combined with a monthly exchange transfusion program in the three patients, which was switched to hydroxyurea in one because of delayed hemolytic transfusion reaction.

### 3.4. Acute Sickle Cell Hepatic Crisis, Sickle Cell Intrahepatic Cholestasis, and Acute Hepatic Sequestration

The combined prevalence of these complications was about 6% (Figure 2).

Twenty seven children presented 28 episodes of acute sickle cell hepatic crisis (one child had two episodes at a 4-year interval). Acute sickle cell hepatic crises were concomitant with other SCD complications in 14 cases (7 acute chest syndromes, 5 painful crises, 1 acute splenic sequestration, and 1 pneumococcal meningitis). Four children were undergoing monthly exchange transfusion for prevention of recurrent stroke or pain, with an HbS level being 15%, 20%, 20% and 45%, and three received deferasirox. Emergency transfusion or exchange transfusion was performed for 20/28 episodes.

Three patients experienced sickle cell intrahepatic cholestasis, with liver failure (prothrombin time expressed as a percentage of the standard value: 14%, 30%, and 39%; normal >70%), in two cases encephalopathy (one patient required mechanical ventilation) and hyperammoniemia (240–338 µM), and renal failure in one. Two patients were undergoing chronic exchange transfusion for stroke prevention (HbS: 25% in one case, not available in the other). One patient on deferasirox had received three-fold the prescribed dosage, 100 mg/kg/d, for 2 weeks. All three patients received exchange transfusion by erythrocytapheresis and recovered completely in a few days. One had a milder relapse 2 weeks later and underwent a second exchange transfusion, with a favorable outcome.

Eleven children presented acute hepatic sequestration. Median age was low (6.3 years) and no patient had experienced any vaso-occlusive crisis in the last year (Table 1). Hepatic sequestration was associated with other symptoms in 7 cases (2 painful crises, 2 splenic sequestrations, 1 acute chest syndrome, 1 gastroenteritis, and 1 respiratory infection). ALT and γ-GT levels were significantly lower than for children with acute hepatic crisis (*p* = 0.04 and *p* = 0.03 respectively) but liver tests were abnormal in 8 of 11 patients and 3 patients had severe cholestasis (Table 2). One patient had liver failure (prothrombin time 25%) and renal failure and died despite urgent exchange transfusion. One 6-month-old baby with concomitant liver and splenic sequestration experienced cardio-respiratory arrest and survived after resuscitation and emergency red blood cell transfusion. One patient had transient renal failure. Nine of these 11 patients received simple or exchange transfusion.

### 3.5. Transfusion Iron Overload

Eighteen patients had iron overload from chronic transfusions, indicated in 17 for stroke prevention and in one for recurrent splenic sequestration. Prevalence of this complication was about 3%, while 14% of patients were undergoing chronic transfusion (Figure 2). Median serum ferritin level was 3058 ng/mL (956–8500; IQR 1617–4019). None had severe iron overload as assessed by MRI, with median LIC 6.4 mg/g (2.5–8.1; IQR 5.6–7.2). All patients were on chelation with deferasirox.

### 3.6. Hepatotoxicity of Oral Chelators

Hepatotoxicity of deferasirox, the only oral chelator used in this series, was suspected in 5 patients. One child had received three times the appropriate dose because of the mother’s misunderstanding, and intrahepatic sickle cell cholestasis developed (*supra*). Encephalopathy, liver and renal failure rapidly recovered after exchange transfusion. Deferasirox toxicity was also suspected in 4 children, with mostly elevated transaminase levels (Table 2). Deferasirox was suspended in all patients and liver test results returned to the basal level. Deferasirox was progressively reintroduced in 3 patients, without recurrence of biochemical abnormalities. One patient received a bone marrow transplant. Re-challenge was not attempted in the last patient.

### 3.7. Infectious Hepatitis

Ten children had an infection possibly responsible for liver abnormalities. Responsible agents were Epstein Barr virus (*n* = 3), cytomegalovirus (*n* = 1), parvovirus B19 (*n* = 1), chlamydiae and mycoplasma (*n* = 1), hepatitis C virus (*n* = 1) due to transfusion in Africa, hepatitis B virus (*n* = 1) due to transfusion in Africa or horizontal transmission, and an unknown agent (*n* = 2). ALT level was only mildly elevated and normalized rapidly in acute infections (Table 2).

### 3.8. Isolated Liver Tests Abnormalities

Sixteen patients had abnormal liver test results, without other liver symptoms (Figure 2). Five had concomitant lung disease (3 acute chest syndromes), 1 painful crisis and 1 Kikuchi syndrome. One 6-week-old baby presented transient neonatal cholestasis (total bilirubin level 48 µmol/L, conjugated bilirubin level 35 µmol/L, and γ-GT level 164 U/L). Liver test results normalized within a few weeks in 10 children but abnormalities persisted in 6 others without an identified cause (Table 2).

## 4. Discussion

Despite several studies on hepatic complications in adults with SCD [3,4,6,8,15], reports in children are few and are mainly case series [10,11,12]. Here we report hepatobiliary manifestations in a very large cohort of SCD children (*n* = 616), ranging from highly frequent and usually poorly symptomatic cholelithiasis to rare and life-threatening complications. Our university hospital is an expert center in both SCD and hepatology, and most patients included in this study were regularly followed until adulthood.

Nearly 40% of our 616 patients had a history of liver or biliary manifestations. Most had the more severe phenotype HbSS. As expected in a hemolytic disease, many of these symptoms were related to gallstones, which occurred in the oldest children, as previously reported [9]. Although more than half of the gallstones were incidentally discovered, complications occurred in 42% of the documented cases, pain being the most frequent. Other complications, including cholecystitis, cholangitis, and pancreatitis, were potentially severe. Therefore, we considered elective cholecystectomy as indicated even for asymptomatic gallstones. This suggestion follows previous recommendations, based on the reduced morbidity after elective cholecystectomy rather than emergency surgery [16,17]. It was mostly performed through coelioscopy after exchange transfusion. However, surgery is not totally protective because some patients had gallstones after cholecystectomy. Ursodeoxycholic acid is not routinely recommended, as it only dissolves cholesterol gallstones.

The most worrying liver complications were severe hepatic crises, associated with liver failure or associated with a decrease in Hb level and called hepatic sequestration. Unfortunately, one child died, but all others recovered, some from an extremely severe condition, after emergency correction of liver anoxia with exchange transfusion. In patients undergoing monthly exchange transfusions, the percentage of HbS was often found to be relatively low, less than 30%, which shows that decreasing HbS is not totally protective. The liver function recovered rapidly after exchange transfusion, even in patients with previously low HbS percentage. An additive toxic role of deferasirox cannot be excluded, especially in a case of documented overdose. However, the patients receiving deferasirox were also those with a severe disease justifying regular transfusion and were more exposed to hepatic complications. In addition, the outcome after exchange transfusion was rapidly favorable, with clinical and biological return to the baseline condition, which would be surprising if deferasirox toxicity alone was responsible for the liver failure.

The reported incidence of hepatic crises is about 10% in adults with SCD [4], which is higher than in our series. The severity of crises in our patients agrees with previous reports [3,4,6,8,11], with a lower mortality in children than adults [10,11], probably because of fewer co-morbidities: iron overload, alcohol, drugs (such as cocaine), or viral hepatitis [18]. However, our patients’ good outcome may also be due to our strategy of early exchange transfusion in all children with hepatic crisis (liver pain and elevated ALT and/or conjugated bilirubin levels), as soon as a biliary complication is excluded. Exchange transfusion was preferred to simple transfusion because it more efficiently decreases HbS percentage, allowing for faster restoration of the blood flow [19].

Cholangiopathy was rare (only 5 cases), but was the second most severe complication, prompting discussion about liver transplantation. Autoimmune diseases seem more frequent in SCD [12,20,21]. Another risk factor was a heterozygous mutation in *MDR3* gene, a condition known to favor the development of gallstones and biliary cirrhosis in adults [22]. However, the young age of our patients and severity of the biliary disease were unusual. Indeed, requiring biliary drainage and liver transplantation in a 9-year-old child with PSC is extremely rare. Because the bile ducts receive blood from terminal arterial branches where sickling could develop, chronic hypoxemia might have worsened the immune-mediated biliary strictures. The benign pancreatic tumor found in another patient was not linked to SCD but required regular controls. The prevalence of biliary disease in SCD could increase with age, because dilation of bile ducts without obstruction was found via endoscopic retrograde cholangiopancreatography (ERCP) in 27% of young adults with SCD and cholestatic jaundice [23]. Also, PSC will probably be more frequently diagnosed in SCD patients because the global incidence of inflammatory bowel disease is increasing and PSC develops in 5% of patients with ulcerative colitis. In addition, we are following two more SCD patients with PSC (7 and 13 years old at diagnosis) diagnosed after the end of this study. The treatment of SCD cholangiopathy depends on the cause: ursodeoxycholic acid in most cases, steroids and azathioprine in overlap syndrome, endoscopic or percutaneous treatment of major strictures, drainage, and ultimately liver transplantation.

The discussion of liver transplantation in our patients was difficult because the published results of liver transplantation in SCD were poor, the operative risks high, the continuation of the transfusion program recommended, and the mid- and long-term results uncertain [24]. The largest recent experience is about 15 SCD adult patients with transplantation for cirrhosis of mixed origin (iron, viral hepatitis, autoimmunity, alcohol, hypoxemia), acute hepatic crisis, or “acute on chronic” liver failure [25,26]. The survival rate was 55% and 44% at 5 and 10 years, respectively, and the neurological complications were frequent. All other reports are cases or very small series. In general, the outcomes were poor in acute liver failure secondary to hepatic crisis because of a high incidence of vascular thrombosis, graft failure and death [6,27]. Once more, the emergency and, in our experience, very efficient treatment in this condition is exchange transfusion, whatever the level of HbS at diagnosis. For indication other than acute liver failure secondary to hepatic crisis, the results of liver transplantation have improved in recent years because of better selection and preparation of candidates, without SCD-linked co-morbidities, and with aggressive exchange transfusions to maintain a low level of HbS [6]. We emphasize the difficulties of the procedure, which should be performed only in units specialized in both SCD and transplantation, with facilities for exchange transfusion at any time. Liver transplantation does not cure SCD, and sickle crises can relapse, especially in the liver [28]. Moreover, drug toxicities may add to the neurological and renal morbidities of SCD. Therefore, exchange transfusions should be maintained at least in the medium term, together with chelators, preferably deferoxamine, which has no reported hepatotoxicity.

Iron overload was suggested as the cause of abnormal liver test results in 3% of the children followed in our center, while 14% were undergoing chronic transfusion. The only abnormality on liver MRI was mildly increased iron content, and no patient had cardiac overload, as previously reported [29]. Thus, liver toxicity of transfusions was considered mild, although we did not perform confirmatory biopsies. In an autopsy series of 141 patients who died at a mean of 36 ± 11 years of age, Darbari et al. found 16 patients (11%) with cirrhosis and 10 with iron overload; 7 of the 16 (44%) with cirrhosis had iron overload as compared with 3 of 125 (2%) without cirrhosis [30]. This finding strongly supports the need for iron chelation [5], notwithstanding the potential hepatotoxicity of chelators.

Deferasirox was probably responsible for elevated transaminase levels in some patients. As discussed earlier, its role in liver failure cannot be excluded. However, it is usually well tolerated in children, as reported in the ENTRUST study of 267 children with transfusion hemosiderosis, among whom five patients only had increased ALT suspected to be related to deferasirox [31]. The prevalence of this complication was even lower in our cohort, less than 1%.

Infections were not frequent in our cohort, and the prevalence of hepatitis B and C virus infection was particularly low in these children originating from endemic countries. However, most were born in France and had undergone early vaccination against hepatitis B virus. Systematic screening of blood donations has greatly reduced the incidence of post-transfusion viral hepatitis in well- resourced countries [32], but the incidence remains high in low-resource ones [33].

Some patients had isolated abnormal liver test results. However, the complications listed above may be diagnosed later, and specific follow-up is needed. We did not perform liver biopsy if no treatable cause was suspected (such as autoimmune liver disease) because of its high risk in SCD patients. In a series of 14 patients, five (36%) had serious bleeding, and four died [34]. However, for some patients of this series, the biopsy had been performed during an acute hepatic crisis, which is an absolute contraindication. If a liver biopsy is necessary in another situation, transfusion must be organized before the procedure.

Our retrospective study has some limitations; indeed, mistakes in the data warehouse reporting are possible, and files may have been incomplete, although all liver test results were available and all medical records were thoroughly reviewed. Four patients were lost to follow-up. However, our cohort is quite representative of children with SCD, notwithstanding a bias toward patients with severe disease.

Based on our findings, we recommend the following diagnosis and therapeutic strategies.

Liver tests (mainly ALT and total and conjugated bilirubin) should be performed two times a year. First-line physicians should be regularly reminded that elevated conjugated bilirubin level is a sign of liver disease, clinically associated with dark urine, and that both total and conjugated bilirubin levels should be measured, especially in acute situations. Abdominal ultrasonography should be performed once a year from age 5, and elective cholecystectomy should be proposed in patients with incidentally discovered gallstones. Liver MRI should be performed every year in children older than 5 years under a monthly transfusion program, in order to assess LIC.

**Figure 3:**
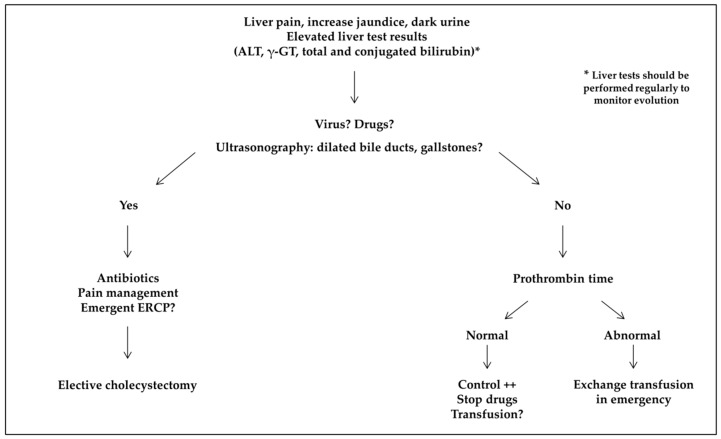
Screening for acute hepatobiliary complications. ALT: alanine aminotransferase; ERCP: endoscopic retrograde cholangiopancreatography; γ-GT: gamma-glutamyltransferase.

For acute conditions, liver tests (ALT, total and conjugated bilirubin, and γ-GT) are not discriminant for the cause but should be performed regularly to monitor evolution (Figure 3). In patients presenting acute abdominal pain and elevated liver test results, ultrasonography should be performed in an emergency to rule out a complication of gallstones, coagulation monitored, and specific tests for acute viral hepatitis obtained (cytomegalovirus, Epstein-Barr virus, and hepatitis A, E, and B virus if the patient was not vaccinated). If the prothrombin time is abnormal, together with increased conjugated bilirubin and ALT levels, exchange transfusion should be organized in an emergency. Exchange transfusion should also be performed if the conjugated bilirubin level is >50 µmol/L (3 mg/L). Simple transfusion should be discussed in other cases. In our series, HbS percentage was not related to the severity of acute hepatic complications, and liver failure developed in children on an exchange transfusion program.

Some of our patients experienced a sickle crisis in the liver as well as in other organs, which could be misleading. ALT and conjugated bilirubin levels should be monitored in any child with abdominal crisis or acute chest syndrome, especially those with increasing jaundice.

**Figure 4:**
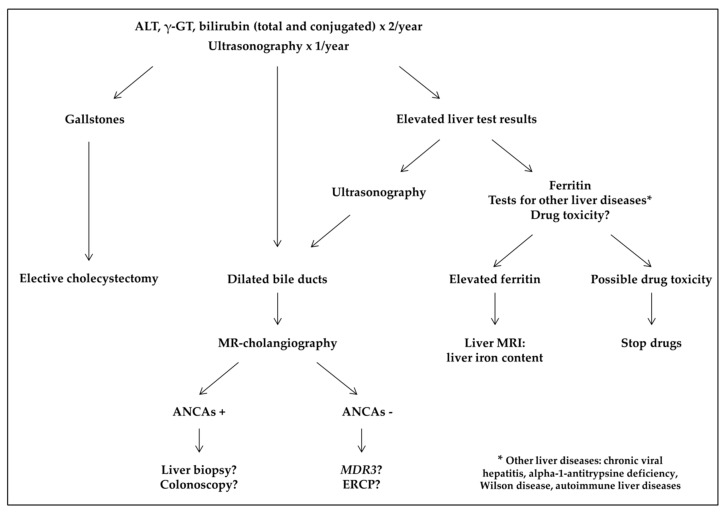
Screening for chronic hepatobiliary complications. ALT: alanine aminotransferase; ANCAs: antineutrophil cytoplasmic antibodies; ERCP: endoscopic retrograde cholangiopancreatography; *MDR3*: multidrug resistance protein 3; γ-GT: gamma-glutamyltransferase.

In chronic conditions, in patients with dilated bile ducts, MR-cholangiography should be performed and autoantibodies for autoimmune liver diseases detected if biliary strictures are seen (Figure 4). In case of positivity for ANCAs, colonoscopy should be discussed, depending on symptoms. If autoantibodies are not found, an ERCP should be discussed, looking for an obstruction not seen on MRI, as it was the case for one of our patients. A genetic susceptibility to cholestasis could also be explored, with a gene panel or *MDR3* only, depending on the local expertise.

When transaminase levels are chronically elevated, specific tests should rule out chronic viral hepatitis, alpha-1-antitrypsine deficiency, Wilson disease, and autoimmune liver diseases. Unnecessary drugs should be suspended. When deferasirox toxicity is suspected, the drug should be stopped until normalization of liver test results. If liver test results return to normal, secondary challenge with deferasirox could be discussed, initially at a lower dose. If ALT level increases again, another chelator should be used. Liver biopsy could be discussed but always with caution and after an exchange transfusion.

## 5. Conclusions

In conclusion, liver and biliary diseases were frequent in SCD children from our large cohort. Cholelithiasis was the most frequent (25%) and was responsible in some cases for severe complications, which could be prevented with elective cholecystectomy. The rare but potentially extremely severe hepatic crises should be suspected in every child with liver pain, increased jaundice and dark urine. Simple biology tests and ultrasonography performed in an emergency were sufficient for diagnosis. In most cases, emergency treatment with exchange transfusion prevented or treated acute liver failure. An additive role for deferasirox could be suspected. Transfusion iron overload was not yet a clinical problem in our pediatric patients. Autoimmune liver disease was rare but should be suspected in patients with chronically abnormal liver test results and autoantibodies. The evolution of primary sclerosing cholangitis was surprisingly severe, perhaps due to the associated biliary ischemia. Liver transplantation was discussed only in patients with chronic severe biliary disease and was a difficult procedure. Long-term liver disease in adulthood remains a concern.

## Figures and Tables

**Table 1:** Clinical characteristics of patients with hepatobiliary complications.

	Lithiasis (Screening or Pain)	Migrating Lithiasis	Cholangio-Pathy	Acute Hepatic Crisis	Acute Hepatic Sequestration	Iron Overload	Suspected Chelator Toxicity	Infectious Hepatitis	Isolated Biochemical Abnormalities
*N*	137	19	5	31 *	11	18	5	10	16
Age (years)	10.4 (2.5–20; 7.5–12.5)	10.9 (4.9–19.3; 8.2–13.4)	9.9 (7.6–14.6; 8.8–11.3)	12 (1.1–17.6; 6.3–13.8	6.3 (0.7–15.4; 4.9–9.6)	11.5 (4.3–16.9; 8.1–14.5)	4.9 (3.2–14.3; 4.6–9.9)	6.8 (2.8–17.6; 4.2–10.6)	9.9 (2.3–16.9; 8.3–12.2)
Female (%)	69/137 (50%)	7/19 (37%)	3/5 (60%)	13/31 (42%)	5/11 (45%)	9/18 (50%)	1/5 (20%)	4/10 (40%)	8/16 (50%)
Genotype (SS-S/β_0_- S/β_+_-SC)	124-5- 3-5	17-0- 0-2	5-0- 0-0	28-2- 1-0	10-0- 0-1	18-0- 0-0	5-0- 0-0	9-0- 0-1	16-0- 0-0
G6PD deficiency (%)	18/115 (16%)	3/17 (18%)	1/4 (25%)	2/28 (7%)	4/10 (40%)	3/13 (23%)	0/2 (0%)	0/10 (0%)	1/12 (8%)
VOC (nb/year)	0.9 ± 1.6	1.6 ± 1.6	0.2 ± 0.4	0.9 ± 1.4	0.0 ± 0.0	0.6 ± 1.7	0.2 ± 0.4	0.4 ± 0.7	1.6 ± 3.1
HU (%)	40/137 (29%)	10/19 (53%)	4/5 (80%)	15/31 (48%)	2/11 (18%)	2/18 (11%)	0/5 (0%)	2/10 (20%)	7/16 (44%)
MET (%)	19/137 (14%)	0/19 (0%)	2/5 (40%)	6/31 (19%)	0/11 (0%)	16/18 (89%)	5/5 (100%)	3/10 (30%)	1/16 (6%)

Data are median (range; interquartile range (IQR)), mean ± SD or percentage. HU: hydroxyurea; MET: monthly exchange transfusions; Nb: number; VOC: vaso-occlusive crisis. * 28 acute sickle cell hepatic crises and 3 sickle cell intrahepatic cholestasis (total = 31 acute hepatic crises). Only 3 patients had autoimmune hepatitis, 2 of them being reported in the “cholangiopathy” group. “Acute hepatic crisis” includes acute sickle cell hepatic crisis and sickle cell intrahepatic cholestasis.

**Table 2:** Biological characteristics of patients with hepatobiliary complications.

	Lithiasis (Screening or Pain)	Migrating Lithiasis	Cholangiopathy	Acute Hepatic Crisis	Acute Hepatic Sequestration	Iron Overload	Suspected Chelator Toxicity	Infectious Hepatitis	Isolated Biochemical Abnormalities
*N*	137	19	5	31	11	18	5	10	16
Hb (g/dL)	8.3 (5.1–12.3; 7.3–9.5)	8.8 (5.5–11.8; 7.1–9.7)	8.9 (6.6–9.7; 8.4–9.2)	8.5 (4.1–11.6; 7.2–9.2)	5.5 (3.9–8.0; 4.9–6.1)	NA	NA	7.0 (6.3–9.3; 6.5–8.8)	8.6 (6.3–9.6; 8.0–9.0)
ALT (U/L)	24 (8–210; 17–32)	105 (17–384; 71–287)	33 (25–109; 25–96)	134 (36–1624; 78–297)	53 (16–213; 47–183)	25 (13–204; 17–32)	303 (73–1624; 194–459)	65 (18–250; 27–238)	60 (15–255; 36–112)
Total bilirubin (µmol/L)	47 (7–161; 30–70)	112 (15–375; 56–150)	50 (33–409; 34–57)	54 (13–685; 27–118)	56 (21–570; 32–241)	47 (20–171; 35–75)	27 (22–40; 25–31)	27 (16–52; 22–37)	52 (12–224; 39–70)
Conjugated bilirubin (µmol/L)	9 (3–63; 8–12)	37 (6–292; 10–83)	24 (12–331; 14–31)	21 (5–465; 11–64)	20 (7–430; 12–185)	10 (5–16; 7–10)	9 (6–30; 7–17)	11 (6–15; 9–14)	13 (7–23; 9–15)
γ-GT (U/L)	20 (5–19; 16–30)	120 (15–432; 53–216)	115 (19–496; 44–149)	169 (14–780; 40–264)	32 (17–162; 20–64)	17 (12–115; 15–24)	43 (14–180; 28–86)	37 (18–143; 35–50)	50 (15–136; 38–70)

Data are median (range; IQR). ALT: alanine aminotransferase; Hb: hemoglobin. NA: not applicable; γ-GT: gamma-glutamyltransferase. “Acute hepatic crisis” includes acute sickle cell hepatic crisis and sickle cell intrahepatic cholestasis.
